# Meeting sustainable development goals via robotics and autonomous systems

**DOI:** 10.1038/s41467-022-31150-5

**Published:** 2022-06-21

**Authors:** Solène Guenat, Phil Purnell, Zoe G. Davies, Maximilian Nawrath, Lindsay C. Stringer, Giridhara Rathnaiah Babu, Muniyandi Balasubramanian, Erica E. F. Ballantyne, Bhuvana Kolar Bylappa, Bei Chen, Peta De Jager, Andrea Del Prete, Alessandro Di Nuovo, Cyril O. Ehi-Eromosele, Mehran Eskandari Torbaghan, Karl L. Evans, Markus Fraundorfer, Wissem Haouas, Josephat U. Izunobi, Juan Carlos Jauregui-Correa, Bilal Y. Kaddouh, Sonia Lewycka, Ana C. MacIntosh, Christine Mady, Carsten Maple, Worku N. Mhiret, Rozhen Kamal Mohammed-Amin, Olukunle Charles Olawole, Temilola Oluseyi, Caroline Orfila, Alessandro Ossola, Marion Pfeifer, Tony Pridmore, Moti L. Rijal, Christine C. Rega-Brodsky, Ian D. Robertson, Christopher D. F. Rogers, Charles Rougé, Maryam B. Rumaney, Mmabaledi K. Seeletso, Mohammed Z. Shaqura, L. M. Suresh, Martin N. Sweeting, Nick Taylor Buck, M. U. Ukwuru, Thomas Verbeek, Hinrich Voss, Zia Wadud, Xinjun Wang, Neil Winn, Martin Dallimer

**Affiliations:** 1 Sustainability Research Institute, School of Earth and Environment, University of Leeds, Leeds, UK; 2 Institute of Landscape Planning and Ecology, University of Stuttgart, Stuttgart, Germany; 3 School of Civil Engineering, University of Leeds, Leeds, UK; 4 Durrell Institute of Conservation and Ecology (DICE), School of Anthropology and Conservation, University of Kent, Canterbury, UK; 5 Department of Environment and Geography, University of York, York, UK; 6 Department of Population Medicine, College of Medicine, QU Health, Qatar University, Doha, Qatar; 7 Centre for Ecological Economics and Natural Resources, Institute for Social and Economic Change, Bengaluru, India; 8 Sheffield University Management School, University of Sheffield, Sheffield, UK; 9 Department of Pharmacology, St John’s Medical College and Hospital, Bengaluru, India; 10 Department of Urban Studies and Planning, University of Sheffield, Sheffield, UK; 11 Smart Places, Council for Scientific and Industrial Research, Pretoria, South Africa; 12 Industrial Engineering Department, University of Trento, Trento, Italy; 13 Advanced Wellbeing Research Centre, Sheffield Hallam University, Sheffield, UK; 14 Department of Chemistry, Covenant University, Ota, Nigeria; 15 Department of Civil Engineering, School of Engineering, University of Birmingham, Birmingham, UK; 16 School of Biosciences, University of Sheffield, Sheffield, UK; 17 School of Politics and International Studies, University of Leeds, Leeds, UK; 18 Department of Automatic Control and Micro-Mechatronic Systems, Femto-st Institute, Besançon, France; 19 Department of Chemistry, University of Lagos, Lagos, Nigeria; 20 Universidad Autónoma de Querétaro, Querétaro, Mexico; 21 School of Mechanical Engineering, University of Leeds, Leeds, UK; 22 Centre for Tropical Medicine and Global Health, Nuffield Department of Medicine, University of Oxford, Oxford, UK; 23 Department of Computer Science, University of York, York, UK; 24 Department of Architecture, Notre Dame University-Louaize, Zouk Mosbeh, Lebanon; 25 WMG, University of Warwick, Coventry, UK; 26 College of Natural and Computational Sciences, University of Gondar, Gondar, Ethiopia; 27 Digital Cultural Heritage Research Center, Sulaimani Polytechnic University, Sulaymaniyah, Iraq; 28 Department of Physics, Covenant University, Ota, Nigeria; 29 Global Food and Environment Institute, School of Food Science and Nutrition, University of Leeds, Leeds, UK; 30 Department of Plant Sciences, University of CA, Davis, MA USA; 31 School of Natural and Environmental Sciences, Newcastle University, Newcastle upon Tyne, UK; 32 School of Computer Science, University of Nottingham, Nottingham, UK; 33 Central Department of Geology, Tribhuvan University, Kathmandu, Nepal; 34 Department of Biology, Pittsburg State University, Pittsburg, KS USA; 35 School of Electronic and Electrical Engineering, University of Leeds, Leeds, UK; 36 Department of Civil and Structural Engineering, University of Sheffield, Sheffield, UK; 37 MB Rumaney Scientific Consulting, Cape Town, South Africa; 38 SADC Centre for Distance Education, Botswana Open University, Gaborone, Botswana; 39 International Maize and Wheat Improvement Center, ICRAF, Nairobi, Kenya; 40 FAIR-SPACE, Surrey Space Centre, University of Surrey, Guildford, UK; 41 The Urban Institute, Faculty of Social Science, University of Sheffield, Sheffield, UK; 42 Department of Food Science and Technology, Federal Polytechnic Idah, Idah, Nigeria; 43 HEC Montreal, Montreal, QC Canada; 44 Institute for Transport Studies and School of Chemical and Process Engineering, University of Leeds, Leeds, UK; 45 Department of Environmental Design, School of Art and Design, Changzhou Institute of Technology, Changzhou, China

**Keywords:** Sustainability, Civil engineering

## Abstract

Robotics and autonomous systems are reshaping the world, changing healthcare, food production and biodiversity management. While they will play a fundamental role in delivering the UN Sustainable Development Goals, associated opportunities and threats are yet to be considered systematically. We report on a horizon scan evaluating robotics and autonomous systems impact on all Sustainable Development Goals, involving 102 experts from around the world. Robotics and autonomous systems are likely to transform how the Sustainable Development Goals are achieved, through replacing and supporting human activities, fostering innovation, enhancing remote access and improving monitoring. Emerging threats relate to reinforcing inequalities, exacerbating environmental change, diverting resources from tried-and-tested solutions and reducing freedom and privacy through inadequate governance. Although predicting future impacts of robotics and autonomous systems on the Sustainable Development Goals is difficult, thoroughly examining technological developments early is essential to prevent unintended detrimental consequences. Additionally, robotics and autonomous systems should be considered explicitly when developing future iterations of the Sustainable Development Goals to avoid reversing progress or exacerbating inequalities.

## Abstract (web summary)

A horizon scan was used to explore possible impacts of robotics and automated systems on achieving the UN Sustainable Development Goals. Positive effects are likely. Iterative regulatory processes and continued dialogue could help avoid environmental damages and increases in inequality.

## Introduction

**Fig. 1:**
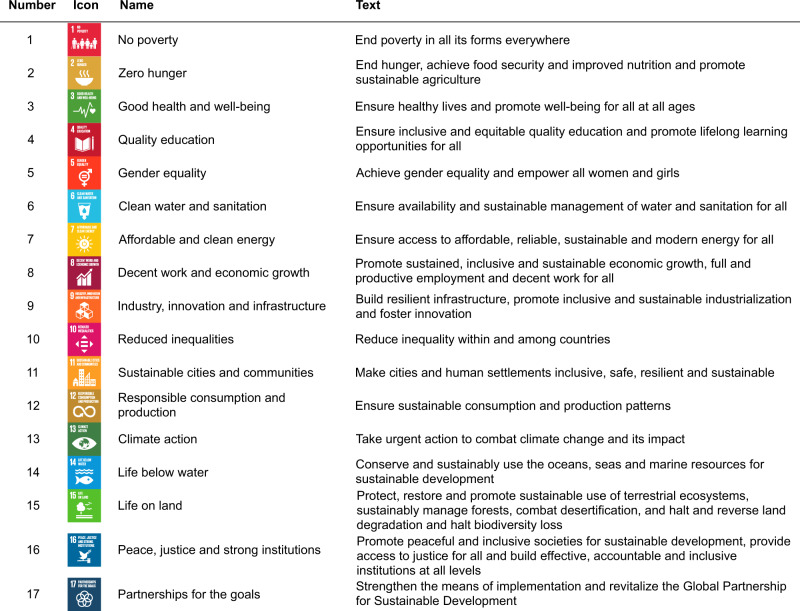
The 17 Sustainable Development Goals as presented by the United Nations^1^ (https://www.un.org/sustainabledevelopment/), with their numbers, icons, titles and full text. Descriptions of the targets are given in Supplementary Figs. 3–11. The content of this publication has not been approved by the United Nations and does not reflect the views of the United Nations or its officials or Member States.

The Sustainable Development Goals (SDGs) were developed as an internationally agreed “plan of action for people, planet and prosperity”^1^. The 17 goals (Fig. 1) and 169 targets cover a wide range of ideals, from ending poverty and improving water sanitation to promoting peace, justice and strong institutions^1^. Many of the targets are interconnected with the possibility of co-benefits, but there is also potential for trade-offs, where the progress towards one SDG might hinder progress towards another^2^. Meeting the SDGs will require investments across society^3–5^, combining government-, civil society- and private sector-led actions^1,3,6^. As of early 2020, insufficient progress was being made towards meeting the SDGs by 2030^1^. For instance, actions were still needed to curtail inequalities within and between countries, reduce hunger or lower carbon emissions^7^. The coronavirus pandemic has also stalled some previous progress by, for example, pushing an extra 124 million people into poverty and exacerbating health inequalities^7^.

Technological advancements have profoundly altered how economies operate and how people, society and environments inter-relate. A critical innovation is the emergence of robotics and automatic systems (RAS)^8^, with the ability to sense, analyse, interact with and manipulate their physical environment with minimal human intervention^9^. Globally, RAS are projected to be adopted by 60% of companies by 2025^10^. Their deployment is expected to change decision making-processes and the way humans interact with one another, governments, and the environment^11,12^.

Mobilising digital technology, such as RAS, could significantly facilitate the achievement of the SDGs^8^. For instance, artificial intelligence has the potential to enable delivery of 134 SDG targets across all SDGs, through mechanisms such as supporting resource efficiency in smart cities and improving modelling of climate change impacts^12^. SDGs can also be inhibited by artificial intelligence, with 59 targets impacted, particularly those centred on poverty, education and inequalities^12^. The limited information that is available regarding how RAS may impact the SDGs tends to centre on individual SDGs. Positive impacts include how RAS can improve health through surgical procedures enhancements^13^ and integrated nursing care^14^, transform agriculture through changes in weed control practices^15^, and contribute to biodiversity conservation through the control of invasive species^16–18^. There is also some concern about how RAS could change the job market^19,20^, influence pollution and waste^21^, be detrimental to biodiversity conservation by directly replacing living components of nature, such as pollinators^22^, and could increase carbon emissions from transport if implemented too widely^23^. Additionally, we have no systematic understanding of how RAS may impact society and the environment, nor how they might facilitate or impede the delivery of the SDGs as a whole. Indeed, plans to address SDGs rarely account for the potential of RAS which, in turn, are developed with little consideration of the SDGs^24^.

**Fig. 2:**
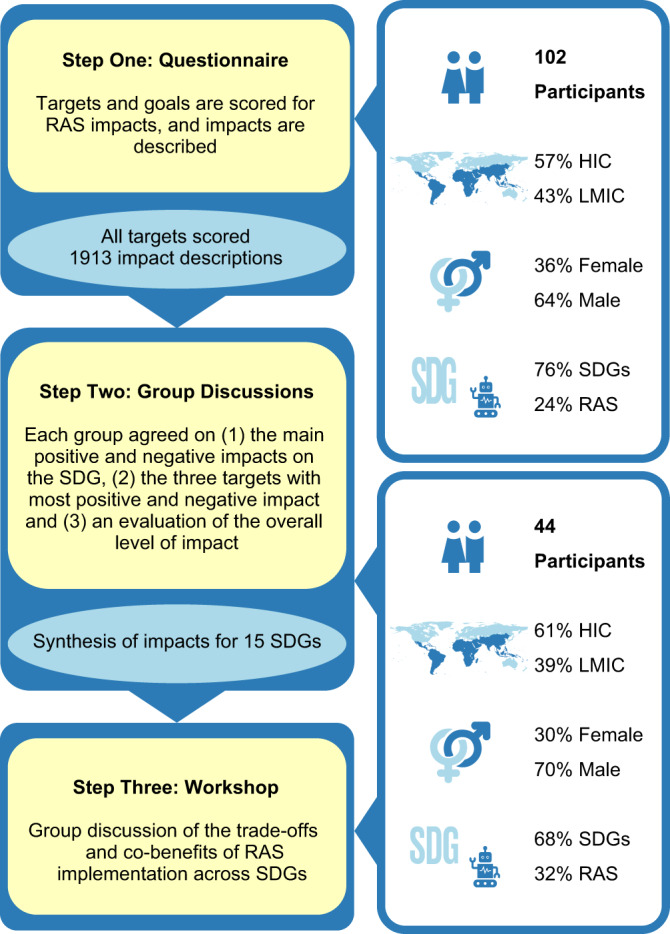
Horizon scan process used to identify the opportunities and threats from robotics and autonomous systems (RAS) deployment for the Sustainable Development Goals (SDG). The horizon scan comprised of a three steps process including an online questionnaire, a group synthesis exercise and a workshop. HIC high-income countries, LMIC low-and middle-income countries.

Here we report the findings of an online horizon scan to evaluate the future key opportunities and threats associated with RAS in relation to all SDGs, as well as the potential for co-benefits and trade-offs among different SDGs linked to RAS implementation. Horizon scans are not conducted to fill knowledge gaps in the conventional research sense but are used to explore emerging trends and developments with the intention of fostering innovation and facilitating proactive responses by researchers, managers, policymakers and other stakeholders^25^. Using a structured and iterative survey (Fig. 2), designed to involve a large range of participants and a diversity of perspectives, we systematically collated and synthesized knowledge from 102 experts. The experts were based in 23 countries and their combined research expertise was global in scope (Supplementary Fig. 1).

## Results and discussion

**Fig. 3:**
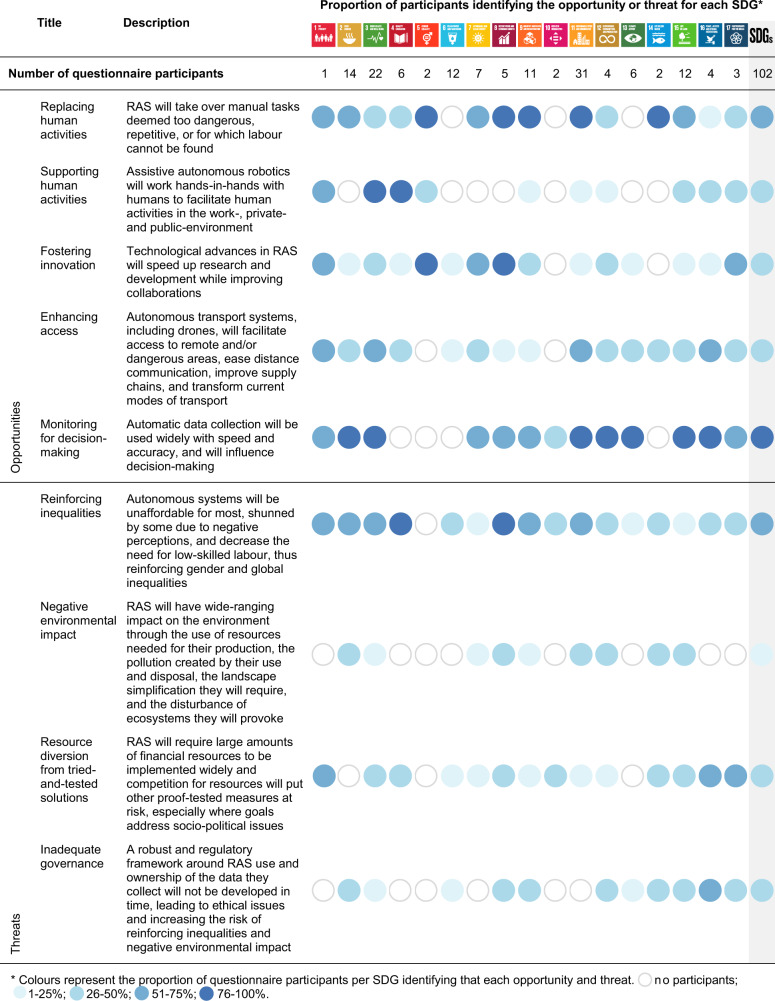
Description of each opportunity and threat, with proportion of questionnaire participants identifying each, highlighting the level to which each opportunity or threat is considered relevant for the Sustainable Development Goal (SDG). See Fig. 1 and https://www.un.org/sustainabledevelopment/ for SDG icon definitions and further information. The content of this publication has not been approved by the United Nations and does not reflect the views of the United Nations or its officials or Member States.

Through content analysis of an online questionnaire (102 participants), group synthesis and workshop content (44 participants), we identified five key opportunities and four key threats (Fig. 3) that need to be considered while developing, deploying and governing RAS with respect to achieving or impeding the achievement of SDGs. We then quantified, based on a Likert scale, the positive and negative impact of RAS on each SDG, as well as the associated uncertainties.

### Key opportunities to meet the SDGs using RAS

Two of the opportunities emphasised how RAS could either (1) replace or (2) support human activities in work, private and public realms. RAS were also deemed to have potential to (3) foster innovation by speeding up research and development, (4) enhance access by transforming transportation systems and enabling safer access to remote areas, and (5) improve monitoring to support and inform decision-making.

Fifty-eight percent of participants noted that autonomous tasks that transform the built and natural environment could contribute to SDGs covered by their expertise (Fig. 3), emphasizing the salience of this opportunity. As such, RAS would be replacing humans in activities that are unsafe, repetitive, or for which workforce recruitment and retention is difficult. Examples given by participants covered crop production;, livestock and fisheries management; processing and packaging; waste and environmental management^26^; eradication of invasive species^18^; treatment of quarantined patients; disinfecting/cleaning public spaces^27^; laboratory work; and manufacturing, construction or repair of built infrastructure^28^, including water management systems^29^. The main advantages over current practices envisaged by the participants were improved infrastructure maintenance, as *“the principles of using RAS in infrastructure are to reduce the size of defect that needs repairing by making frequent small repairs”*, increased productivity, and reduced resource utilisation, potentially making goods and services more sustainable and/or cheaper.

The opportunity for supporting human activities was recognised by 31% of participants (Fig. 3), highlighting that this opportunity was less recognised. Participants highlighted that RAS might decrease human workloads where there is a shortage of workforce, such as in elderly care^14^. In health, participants suggested that RAS would enhance surgical practices^13^ and the physical movement of patients within healthcare facilities^13^. Additionally, they believed RAS might facilitate specific health screening activities, such as sexual health diagnostics, by distancing the human presence and the associated fear of judgment. Participants underlined how RAS could improve education^30^ by offering everybody the opportunity of a quality education and vocational training, personalised according to their needs.

Participants highlighted that RAS could also contribute to supporting specific public and private needs, for example, by providing help in overcoming physical or cognitive limitations. Socially assistive robots such as Nao^31^, a humanoid robot intended to interact with humans in education and healthcare settings, are particularly likely to aid inclusivity by creating a *“large amount of possibilities for physically impaired, autistic or vulnerable people”*, including improving learning skills^32^ and providing a safe, protected environment in which *“RAS can be reliable enabler companions […] and monitoring systems for anyone (including women, children, older persons and persons with disabilities) in public spaces”*.

RAS were perceived by 28% of participants as helping to achieve the SDGs by fostering innovation (Fig. 3). RAS were described as being *“the leading edge of technology development, […] based on the most advanced scientific knowledge and […] developed for solving industrial challenges”*. Participants believed that RAS would speed up the research process across sectors but, in particular, the efficiency in the development of drugs/vaccines and renewable energies. Participants also suggested that RAS-led entrepreneurship could encourage creativity, stimulate the creation of highly skilled jobs^10^, and lessen inequalities between countries through RAS technology transfer.

Forty-six percent of the participants suggested that RAS could contribute to progress towards the SDGs by enhancing access to remote and/or dangerous areas, facilitating interactions at distance and transforming current modes of transport (Fig. 3). Participants noted that enhancing access could have implications for improving disaster relief, for instance, by providing ambulance services. RAS could also help those in remote areas to access basic services, with examples ranging from how “*early childhood remote diagnosis and consultation may reduce mortality”* to delivering medical supplies, blood or vaccines^38^, or improving education^30^. Furthermore, RAS could facilitate environmental conservation and research in inaccessible locations^39^. Even in seemingly accessible locations such as cities, participants thought that RAS could manage features that are otherwise expensive, dangerous or difficult for humans to access, such as vertical farms^40^ or green walls/roofs. The widespread uptake of autonomous vehicles has the potential to make roads safer while reducing the loss of unproductive time while driving^41^, which will impact how cities are planned^42^, with potentially positive implications for human well-being and the urban environment^21^.

RAS are already widely used for automated monitoring and data collection to support decision-making^17,33–36^ and this opportunity was mentioned by 78% of all participants, highlighting its salience (Fig. 3). Participants stated that autonomous monitoring would take place across many sectors including infrastructure^34^, resource distribution, wildlife populations^17,33^, water quality^37^, global financial markets^35^ and illegal fishing^36^. Participants described such advances as critical to *“provide […] a good framework for assisted decision-making, planning and governance”*. Additionally, participants suggested that the collection of big data facilitated by RAS would provide opportunities to “*mak[e] massive public participation in [planning] easy and cost-effective”*. Automated monitoring was felt to be faster, more responsive to change, more transparent and devoid of human errors compared to manual methods. Participants were, however, concerned that *“monitoring* per se *isn’t actually going to deliver [actions towards the SDGs]”*.

### Key threats to achieving the SDGs because of RAS deployment

Four threats were identified that could impede the achievement of the SDGs (Fig. 3), with participants noting that RAS implementation could (1) reinforce inequalities due to a lack of affordability and transformation of the job market, and (2) negatively affect the environment via novel forms of biodiversity disturbance, as well as through the manufacture and disposal of RAS throughout their lifecycle. We also identified concerns that RAS would (3) divert resources away from tried-and-tested approaches to achieving the SDGs. All three of these threats could then be compounded through (4) the inadequate governance of RAS, while also posing ethical issues about data use.

The primary and most salient threat, raised by 51% of participants, was that RAS deployment would reinforce existing inequalities because *“through the course of history […] automation has always had a tendency to ease the accumulation of wealth, typically benefiting those who are already wealthy”*. Participants envisaged scenarios whereby inequalities could be exacerbated by cultural contexts and negative perceptions that communities hold for RAS, such as RAS *“contradict[ing] the ideas of agricultural production embraced by indigenous peoples”* or human interactions being necessary for some occupations such as teaching or nursing. Inequalities could also be intensified by a transformed job market, as the need for low-skilled workers would decrease as *“low skilled, mundane and routine tasks can be automated. Reskilling employees will take time; during which more advanced jobs are probably being ‘taken over’ by RAS”*.

Although the impact of automation on jobs is uncertain^19,20^, the perception of RAS taking over jobs might be sufficient to slow down their deployment in some countries, as *“RAS […] won’t be chosen over […] labour-intensive processes due to loss of livelihood, despite health and productivity benefits”*. Participants thus noted that inequalities might rise between countries, as these negative perceptions interact with different starting points in regards to access to technology^43^, and a greater reliance on primary production and manufacturing rather than services in low- and middle-income countries^44^. However, participants emphasised how negative RAS impacts on the job market could be lessened by *“redefin[ing] what we mean by ‘full and productive employment’ […]. We might consider goals such as ‘full unemployment’ and the encouragement of leisure instead of work”*.

Unless actions are taken^12^, participants felt that RAS were likely to exacerbate existing inequalities by reinforcing pre-existing structural biases. Specifically, if artificial intelligence^45^, which is central to many RAS technologies, is trained on biased datasets and decisions are taken without human intervention, those biases and associated inequalities will be amplified^46^. There are promising ways to mitigate such threats via ensuring biases in datasets are adjusted for using more appropriate algorithms, however those are yet to be tested in the real world and rely on biases in training datasets being openly acknowledged, which is as yet not the norm^47^. Additionally, participants identified the need to empower more women and those from diverse ethnic backgrounds to engage with RAS development. Currently most RAS researchers are male (84%) and white (67%)^48^. This lack of diversity poses a risk that any structural inequalities and pre-existing biases in datasets are unconsciously reinforced by RAS developers who may not fully grasp issues facing minority and underrepresented groups.

Twenty percent of participants were concerned about the potential negative environmental impacts of RAS (Fig. 3). Primarily, these were related to the lifecycle of RAS, including the type and amount of energy required for large-scale RAS deployment^49^, the impact of resource extraction to build RAS and the pollution caused by unrecovered RAS or their disposal^50^. In addition, participants were worried that improvements in productivity catalysed by RAS could well come at the expense of the environment. Landscape simplification is an important driver of environmental change and biodiversity loss^51^. Participants felt that deploying RAS for food production might expand landscape simplification by favouring practices such as sensor-based weed control^52^ and robotic fruit-picking^53^, both of which require relatively simplified landscapes^52,53^. Participants noted how the *“history of the global food system has shown that the use of technology has increasingly contributed to seed poverty [and] environmental devastation”* and were concerned about this being amplified. The negative impact of unmanned aerial vehicles on birds is well-documented^54^. Participants envisaged scenarios where large-scale RAS-deployment would intensify such disruptions and cause comparable issues with other taxonomic groups, including some that are currently poorly known or isolated due to their inaccessible habitats, such as deep-sea organisms^39^.

For many of the SDGs, there are already tried-and-tested approaches that can be used to enhance their delivery. A threat identified by 27% of participants (Fig. 3) was that investments in RAS might divert resources away from more straightforward, less technologically-driven approaches. Participants highlighted that many of the SDGs are *“very human and politically driven ambitions and RAS may not be the best solution to achieve [them]”* and resource allocation to social and political programs were better alternatives (e.g. for achieving SDG10 or SDG16). Participants also warned that investing in technology without similar investment in the social context might be counterproductive. An example of this was *“high-tech public toilet cubicles installed in a city in India as safe and clean units for women to use. However, no-one used them as they were poorly placed and they feared that the automatic door would trap them inside”*. Even for those goals which could benefit from RAS implementation, participants were worried that implementation of RAS systems will be too slow, as *“[t]he current state of the technology is not fully ready […], [r]eliance on autonomy might worsen the situation”*. RAS should not be implemented at the expense of tried-and-tested activities such as vaccination campaigns, education or emergency response services.

Another concern raised by 27% of participants, denoting lower recognition of the threat as compared to the threat of reinforcing inequalities, was the risk of inadequate governance of RAS (Fig. 3). There was consensus that, *“if used wisely”* and fairly, the impact of RAS would be mostly beneficial. Nonetheless, it remains uncertain how RAS-use will be regulated and who might own the resulting data^55^. This raises an important ethical issue, as *“solutions in tech risks creating a form of technological determinism and missing the need for broader reforms, including over who owns and controls tech itself”*. Participants noted that ownership of human behaviour data collected for monitoring related to health, education or institutions could be exploited by transnational companies, authoritarian governments or hackers, with consequences for human rights and privacy^56^. Participants also thought that inadequate governance of RAS could increase the likelihood of reinforcing inequalities or damaging the environment. Participants felt that robust international legal and regulatory frameworks for RAS should promote sharing of intellectual property. If RAS technology and patents are largely owned by transnational companies who can bypass national regulatory frameworks, this might lead to higher operating costs, making RAS unaffordable for most of the population and reinforcing macroeconomic inequalities. Ownership by transnational companies could also augment negative environmental impacts, with participants concerned about the *“possibility that increased automation could further boost large-scale agribusiness that has been degrading ecosystems globally”*.

### Net overall impact of RAS on SDG delivery

**Fig. 4:**
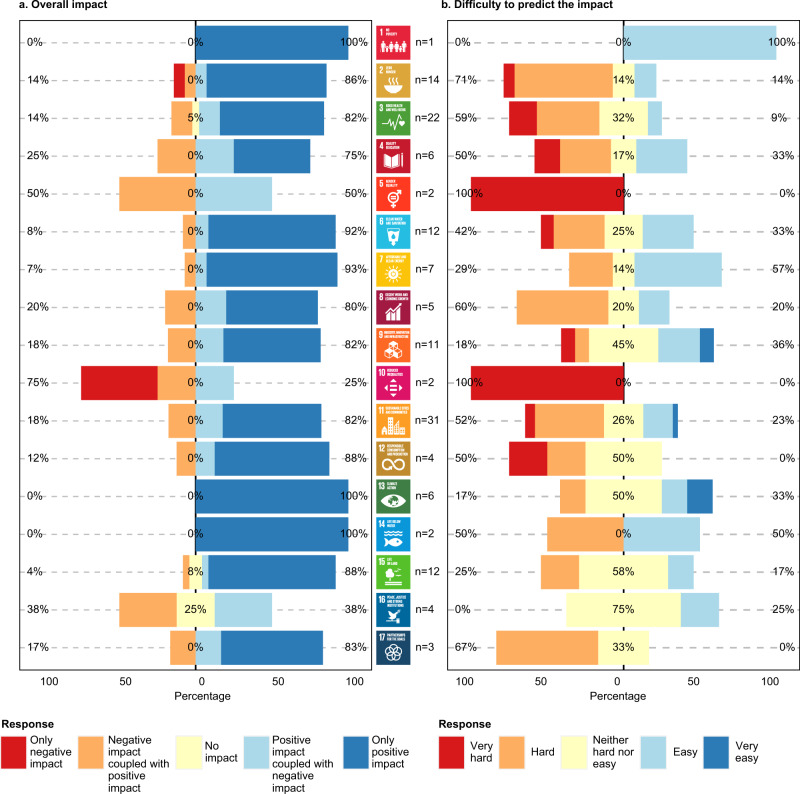
Impact of RAS on the SDGs and the difficulty to predict said impact, as rated by the online questionnaire participants. **a** Overall impact of RAS on the SDGs; **b** Difficulty to predict the impact of RAS on the SDGs. Percentage values indicate the proportion of participants giving negative, neutral and positive scores. See Fig. 1 and https://www.un.org/sustainabledevelopment/ for SDG icon definitions and further information. The content of this publication has not been approved by the United Nations and does not reflect the views of the United Nations or its officials or Member States.

Despite identifying emerging threats, participants indicated that the impact of RAS on progress towards the SDGs was likely to be overwhelmingly positive (Fig. 4a). No SDG was determined to be predominately impacted by RAS negatively, and there were seven SDGs for which more than 75% of the participants believed that RAS would have only positive impacts on their delivery. For the remaining ten SDGs, trade-offs requiring careful management were identified. However, the future net overall impact of RAS on achieving the SDGs was considered hard to predict by most (Fig. 4b), especially for SDGs dealing with inequalities (SDG5 and SDG10). This uncertainty may well reflect the lack of interaction, and hence understanding, between engineering, natural sciences and social sciences experts^57^. Indeed, this was reflected in our participants, none of whom professed RAS expertise alongside knowledge of the SDGs dealing with issues of poverty, equality, justice or institutions (SDG1, SDG5, SDG10, SDG16 or SDG17; Supplementary Fig. 2). The participants only scored the certainty of RAS impacts as ‘very easy’ to evaluate for three SDGs, related to innovation and infrastructure (SDG9), cities (SDG11) and climate (SDG13).

### Co-benefits and trade-offs associated with RAS and the SDGs

Participants identified several SDGs, particularly those associated with environmental issues or multi-dimensional poverty, which would benefit from RAS implementation aligned with meeting other SDGs. For example, RAS implementation for land decontamination, aligned with SDG15, would also contribute to the environmentally sound waste management required to reach SDG12. Reduction of waste produced rather than its management later down the line is also critical in reaching SDG12, and ties closely with *“SDG2 […] in terms of food security, where you could actually deploy RAS [to monitor] consumption and thereby reduce food waste”*. Likely co-benefits of RAS deployments in industry (SDG9) or agriculture (SDG2) were envisaged. For instance, RAS implementation to replace unsafe tasks in agriculture traditionally carried out by women could facilitate improvements in gender equality by decreasing the low paid work burden and freeing up time for education for women (SDG5). Examples with industry include cases when RAS might *“be deployed to gain more transparency across global value chains, and through this further reduce modern slavery aspects globally”*. Due to the strong inter-linkages between health, education and poverty^2^, participants envisaged scenarios whereby the contribution of RAS to education (SDG4) would be a *“determiner of the success of other goals such as gender equality, […] poverty reduction, […] health and the like”*. Equally, RAS-led improvements in food provision (SDG2) or health (SDG3) were thought likely to advance progress towards multi-dimensional poverty reduction (SDG1).

Participants raised concerns regarding trade-offs between the benefits gained by RAS deployment for specific goals, especially where technical solutions may initially be attractive such as in upgrading industries and infrastructure. Opportunities were identified for RAS deployment to increase the efficiency of tasks, but better efficiency and associated cost reductions may well create rebound effects that amplify consumption^58^, thus worsening environmental crises^59^ because more virgin materials would be processed into products each year^2^. Greater efficiency could also further concentrate wealth, reinforcing inequalities, such as in the case of water management, where *“some countries are […] endangering water for others, [so] increasing efficiency for some country might not necessarily be good for another in terms of monopolising water management”*. Trade-offs emerging from RAS deployment were also identified within goals. For instance, the opportunity to enhance access and monitoring was perceived by participants to be as likely to open new routes for over-exploitation as to improve conservation efforts^39^. Some of the trade-offs that were identified are inherent within the SDGs themselves, such as how SDG12, which promotes consumption and production, could lead to trade-offs with SDGs associated with health, poverty reduction and reduced inequalities^2^. Similarly, SDG8 on economic growth has multiple targets that can impede each other^60^.

Participants also felt that over-reliance on RAS for monitoring to support decision-making might undermine progress towards meeting SDGs associated with inclusivity and improved governance, such as SDG11 on sustainable cities and communities and SDG 16 on peace, justice and strong institutions, as *“the decisions made by artificial intelligence algorithm-powered urban planning and management systems will exclude ordinary citizens”*. Increasing use of RAS for informing decision-making could have wide-reaching repercussions by *“trigger[ing] humans to completely delegate the thinking job of decision-making to automated systems, reducing our knowledge and understanding of these complex systems and […] our control on the interplay of these complex factors that are affecting these systems”*.

### RAS and SDGs: ways forward

RAS are here to stay and will fundamentally transform how we interact with one another, technology and the environment^9^. This transformation offers many potential benefits. However, realising those benefits while minimising unintended consequences and trade-offs will be complex. As a starting point, a declared aspiration to contribute positively to the full range of SDGs when designing and deploying RAS^61^ would likely enhance the social and environmental benefits of RAS uptake. Early collaboration and continued dialogue across stakeholders while implementing RAS would contribute to both setting realistic expectations^62^ and helping organisations working for sustainable development^63^ to seize opportunities provided by RAS while avoiding any pitfalls. Greater engagement by engineers with sustainable development professionals would ensure that RAS are developed and deployed while respecting the needs of multiple different groups and mitigating any emerging threats^24,61^ from the outset. Indeed, appropriate mitigation measures to counter the potential negative impacts of RAS would, by their very nature, contribute to addressing the SDGs. For example, improving education would help bridge technological gaps, reducing inequality of access to RAS^20^. Further, strengthening institutions would reduce the likelihood of poor RAS governance. Indeed, strong governance structures are central to mitigating any emerging threats, as is ensuring that adequate regulation is in place prior to widespread uptake will be essential. Robotics are now included in United Nations’ strategies for peace^64^, yet the opportunities and threats posed by RAS are thus far not integrated into any other global initiatives, strategies or social goal setting. In part, this is likely due to the relatively slow pace of regulation and goal setting when compared to RAS development, leaving the door to non-regulation or regulation through non-binding norms or voluntary guidelines^65^. This approach is, however, insufficient as it is unable to ensure inclusivity and representation^65^, which are both pillars of the SDGs. Iterative regulatory processes are that can be adapted in parallel with emerging new technologies are needed to ensure appropriate RAS governance^66^. Although all impacts of RAS across the suite of SDGs are hard to predict, inclusion of RAS in future iterations of the SDGs^67^ will be essential to avoid detrimental and unintended consequences while realising the opportunities they offer.

## Methods

Horizon scans aim to “support the early identification and collective exploration of emerging issues”^68^ through a systematic examination of potential future developments and their related threats and opportunities^69,70^. They can be carried out as either a document-based analysis, focusing on scientific literature, patents or media, or as an expert consultation process^70^. Horizon scans have been used to study a diversity of topics, including bioengineering^71^, security^72^, medicine^73,74^ and biodiversity conservation^21,75^, and are increasingly used by private and public organisations worldwide^69^ to inform decision-making. Here, we conducted a horizon scan of the future potential positive and negative impacts, opportunities and threats that RAS deployment could have on the delivery of the SDGs, and the co-benefits of trade-offs between RAS deployment for using a three-step expert consultation process.

### Horizon scan participants

We adopted a mixed approach to recruitment to minimise the likelihood of bias associated with relying on a single method. We recruited RAS and SDGs experts by directly contacting 1078 people with relevant research profiles. Additional participants were recruited through snowball sampling (i.e. participants suggesting additional experts from their professional networks), mailing lists (e.g. EU robotics, Pipebots) and social media. A pool of 102 participants responded to the online questionnaire, with expertise from across the world (Supplementary Fig. 1) and all SDGs (Supplementary Fig. 2).

We asked participants to describe their areas of expertise, country of employment and countries in which they conduct their work (e.g. research projects, consultancy contracts). Countries were grouped into high-income and low- and middle-income according to the Development Assistance Committee list of official development assistance from the Organisation for Economic Co-operation and Development^76^. Participants were based in 23 different countries, with 58 (57%) in high income and 44 (43%) in low- and middle-income countries (Supplementary Fig. 2g, h). Most participants conducted research, with two working with the private sector, one for a government and one for an NGO. Our participant pool consisted of experts whose primary expertise was in engineering (25%) or mainly aligned with the 17 SDGs (76%) and was 36% female (Supplementary Fig. 2c–f).

Prior to taking part, we asked participants for their consent, informing them that their involvement was voluntary, they could withdraw at any point, and their answers would be anonymised. Ethical approval was granted by the University of Leeds Ethics Research Committee (Reference LTSEE-105). The anonymised quantitative data are available on the University of Leeds Institutional Repository (10.5518/1078). We piloted the questionnaire and, as a consequence, refined some of the wording to improve clarity. The answers from the pilots were discarded and not included in the analysis.

### Horizon scan

The first step of the horizon scan comprised an online questionnaire (Fig. 2) and aimed to evaluate the overall impact of RAS on each SDGs and extract the main opportunities for and threats towards SDG delivery associated with RAS deployment. Given that there are 17 SDGs covering a very broad remit (Fig. 1), we structured the initial questionnaire to allow participants to choose up to three SDGs that best aligned with their expertise. Each SDG covers a wide range of societal issues and consists of several targets (median = 10; range = 5–19; total = 169)^1^. It is likely that the impact of RAS will vary according to the target. We therefore asked participants to evaluate whether RAS would have (a) a positive impact and (b) a negative impact on the achievement of each target of the goal(s) in which they have expertise, using a 5-item Likert scale (strongly disagree, disagree, neutral, agree or strongly agree). A “do not know” category was also included. The response to the Likert scale was used to elicit an in-depth statement to the open-ended follow-up question as to what type of impact(s) were considered, thus providing a description of which opportunities (for positive impacts) or threats (for negative impacts) each achievement of targets would experience from RAS. Participants were also asked to evaluate the impact of RAS on the overall SDG, as either (a) positive, (b) negative, (c) both positive and negative or (d) no impact. The ease of predicting the impact of RAS on each SDG will likely vary according to the level of development/use of RAS in that area. To evaluate the level of certainty associated with RAS impacts on each SDG, we asked the participants to evaluate, through a 5-point Likert scale (very hard, hard, neither hard nor easy, easy or very easy), how difficult they felt it was to predict the impact of RAS on the SDG. The online questionnaire was completed by 102 participants (Supplementary Fig. 2a). Each participant covered a median of one SDG (range = 1–3), providing 144 responses covering all 17 SDGs. Participants submitted 1913 statements across the 169 SDG targets. The statements and quantitative rankings were collated and analysed for each SDG.

For step two of the horizon scan, we wished to synthesise the statements for each SDG. In particular, we wanted to evaluate what the key opportunities and threats associated with RAS deployment for each SDG were, consider whether there was variation in which targets within each SDG were more or less likely to be impacted, and evaluate the overall impact of RAS on the SDG as a whole. To do this, we grouped the participants who opted to continue their participation into 17 groups, assigning them to SDGs that aligned as close to possible with their stated expertise. Each group included at least one engineer. Every group was presented with the collated answers from the first step to carry out the evaluation. Given that step one resulted in a low number of answers for SDG1, the participants assigned to this SDG independently provided their answers to the step one questionnaire prior to the group work. Participants assigned to SDG2 and SDG14 did not contribute. Step two resulted in 15 group synthesis redacted by 44 participants.

Step three of the horizon scan consisted of an online workshop aiming to highlight the interactions, in terms of both co-benefits and trade-offs, between RAS deployed for delivering different SDGs. During this workshop, one representative from each group presented their synthesis. The 44 participants then discussed the interactions with the other SDGs.

### Analysis

We used an inductive approach to content analyse the qualitative data extracted from the in-depth answers from the questionnaire (step one), the synthesis (step two) and the transcriptions from the workshop discussions (step three). Data were grouped according to whether they described (a) an emerging opportunity through which RAS could contribute to the achievement of the SDGs or (b) an emerging threat to take into consideration when designing RAS to avoid any negative impact on the achievement of the SDGs. We took a similar inductive approach to content analysis to extract the co-benefits and trade-offs from the workshop discussions (step three).

We quantified how recognised each opportunity and threat was by calculating the percentage of participants mentioning that opportunity or threat in their in-depth questionnaire answers. We report percentages as an indication of how salient the opportunity or threat was perceived, rather than as a measure of its scientific validity. We generated visualisations of all Likert scores with the “Likert” package^77^ of R, version 4.0.2^78^.

## Supplementary information

Supplementary Information

## Data Availability

The anonymised quantitative dataset generated and analysed during this study is available via the University of Leeds Institutional Repository (10.5518/1078). The qualitative datasets generated and analysed are not publicly accessible due to privacy restrictions.
