# Supplementary material for: Meeting sustainable development goals via robotics and autonomous systems
Source: Nat Commun. 2022 Jun 21;13:3559. doi: 10.1038/s41467-022-31150-5 (PMC9211790; doi:10.1038/s41467-022-31150-5)
Supplement: Supplementary file 1 — Supplementary Information [file 41467_2022_31150_MOESM1_ESM.pdf]

Meeting Sustainable Development Goals via Robotics and Autonomous Systems – Supplementary information

Solène Guenat[1,2,*], Phil Purnell[3], Zoe G. Davies[4], Maximilian Nawrath[1], Lindsay C. Stringer[5], G.R. Babu[6], Muniyandi Balasubramanian[7], Erica E.F. Ballantyne[8], Bhuvana Kolar Bylappa[9], Bei Chen[10], Peta De Jager[11], Andrea Del Prete[12], Alessandro Di Nuovo[13], Cyril O. Ehi-Eromosele[14], Mehran Eskandari Torbaghan[15], Karl L. Evans[16], Markus Fraundorfer[17], Wissem Haouas[18], Josephat Izunobi[19], Juan Carlos Jauregui-Correa[20], Bilal Y. Kaddouh[21], Sonia Lewycka[22], Ana C. MacIntosh[23], Christine Mady[24], Carsten Maple[25], Worku N. Mhiret[26], Rozhen Kamal Mohammed-Amin[27], Olukunle Charles Olawole[28], Temilola Oluseyi[19], Caroline Orfila[29], Alessandro Ossola[30], Marion Pfeifer[31], Tony Pridmore[32], Moti L. Rijal[33], Christine C. Rega-Brodsky[34], Ian D. Robertson[35], Christopher D.F. Rogers[15], Charles Rougé[36], Maryam B. Rumaney[374], Mmabaledi K. Seeletso[38], Mohammed Z. Shaqura[21], L. M. Suresh[39], Martin N. Sweeting[40], Nick Taylor Buck[41], M.U. Ukwuru[42], Thomas Verbeek[43], Hinrich Voss[44], Zia Wadud[45], Xinjun Wang[46], Neil Winn[47], Martin Dallimer[1,*]

* Corresponding authors:

Solène Guenat, University of Stuttgart, Institute of Landscape Planning and Ecology, Keplerstrasse 11, D-70174 Stuttgart, Germany, solene.guenat@ilpoe.uni-stuttgart.de;

Martin Dallimer, Sustainability Research Institute, School of Earth and Environment, University of Leeds, Leeds, LS2 9JT, UK, m.dallimer@leeds.ac.uk.

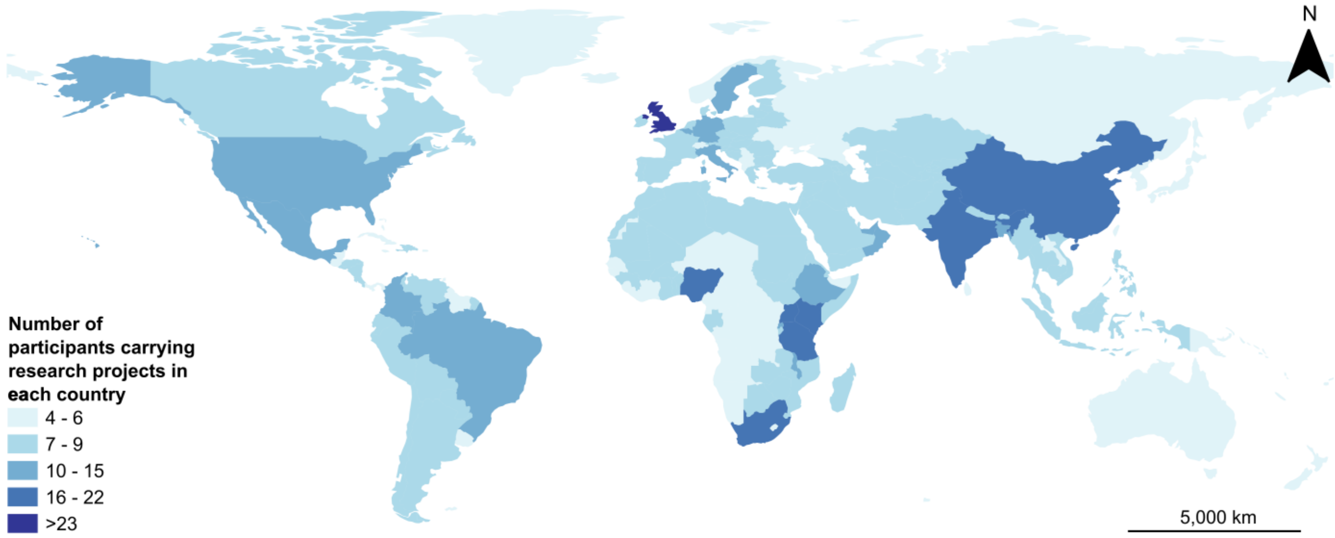

Supplementary Figure 1 Countries in which participants carry out research projects, with darker colours including regions where more participants stated they have expertise. The minimum number of participants in any country is four, as four participants stated they carry out research with a global reach. Map created in QGIS v.3.16, with base maps from Natural Earth.

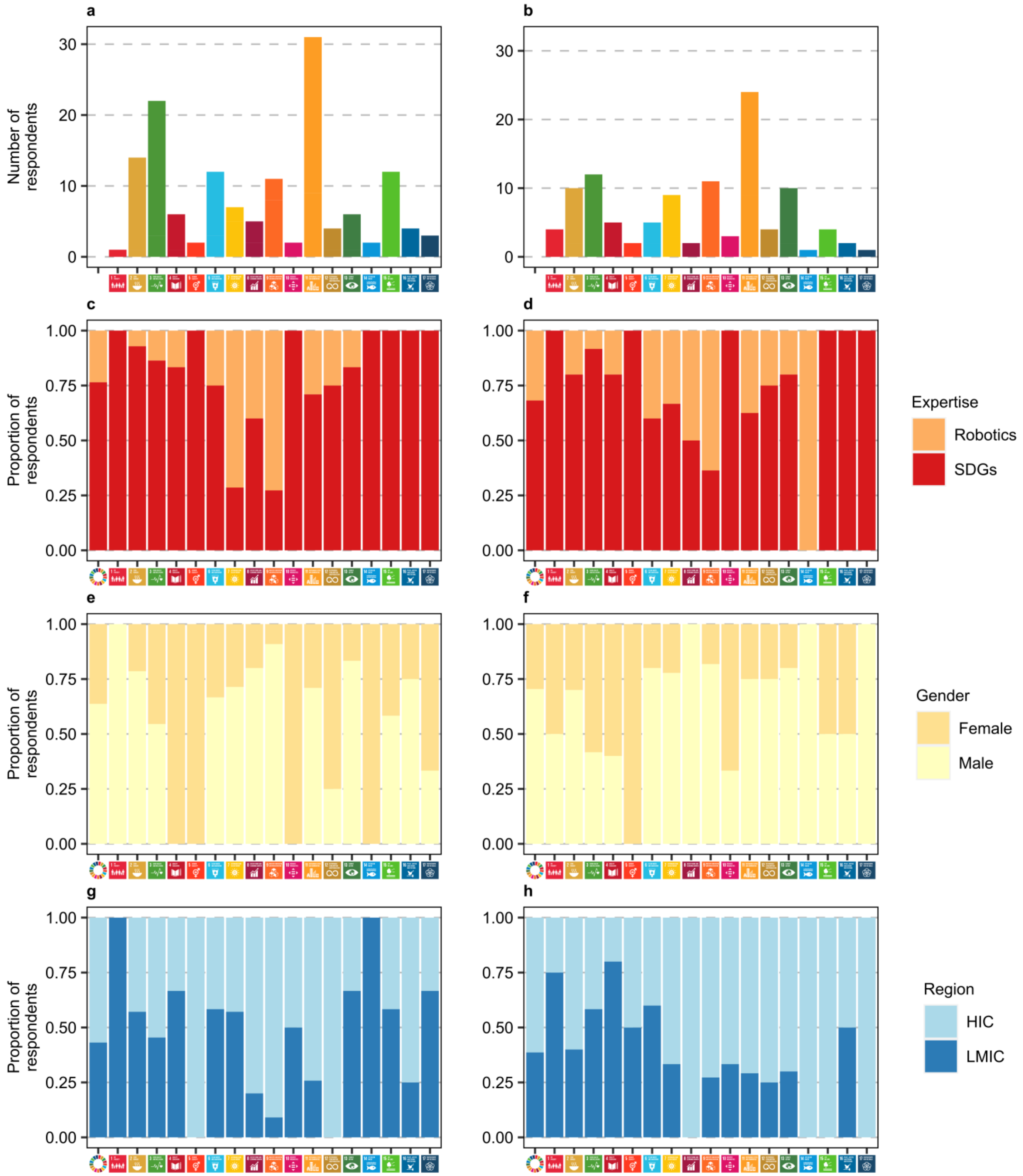

Supplementary Figure 2 Description of the participants. (a-b) Number of participants with expertise in each SDG taking part in the online questionnaire (step one, a) and the group synthesis exercise and workshop (step two and three, b). Participants according to (c-d) their expertise, (e-f) their gender and (g-h) the income category of their region of employment. Values exceed the total number of participants as online questionnaire participants (n=102) could provide responses for several SDGs, according to their expertise. The content of this publication has not been approved by the United Nations and does not reflect the views of the United Nations or its officials or Member States (https://www.un.org/sustainabledevelopment/).

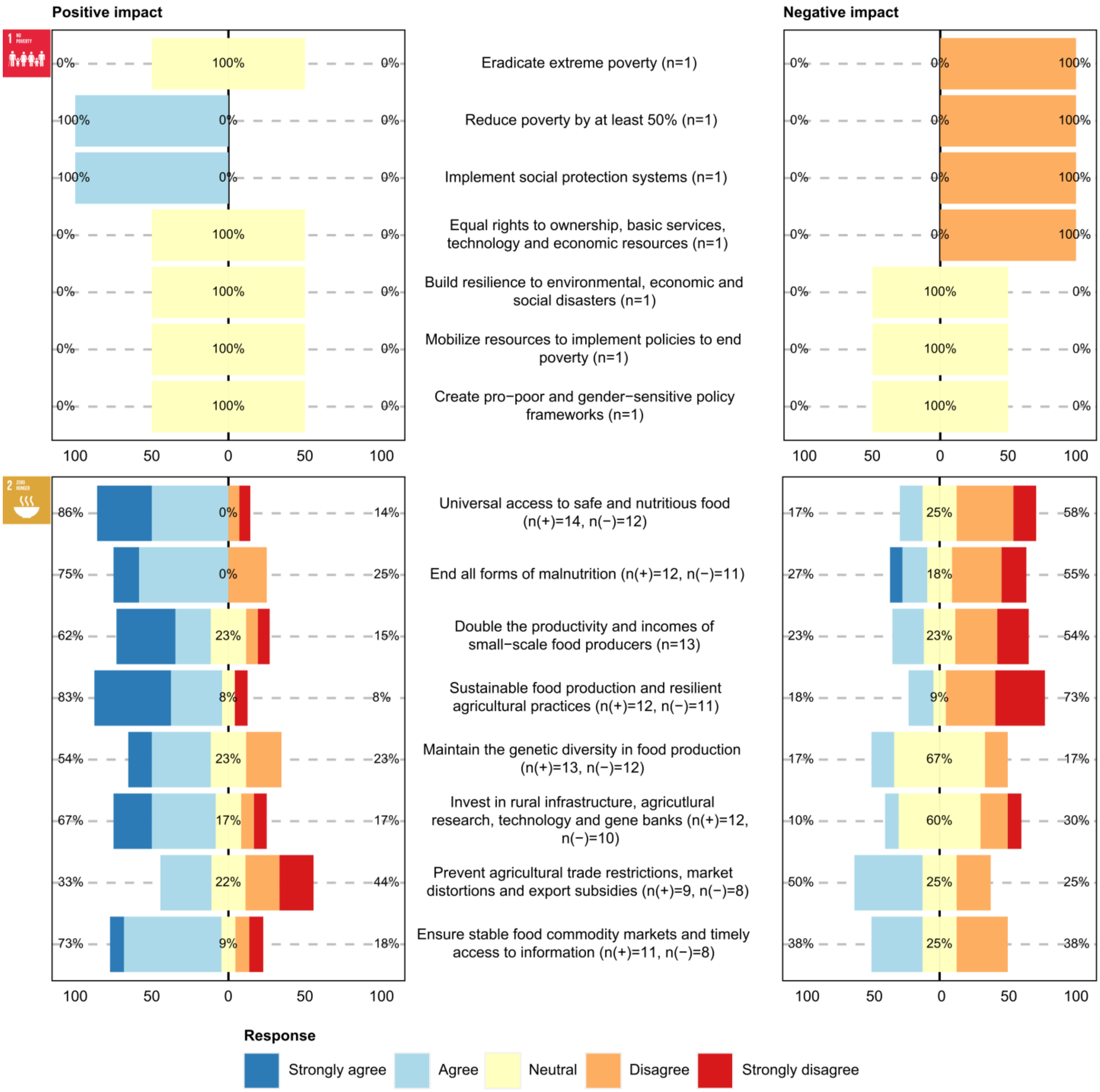

Supplementary Figure 3 Positive and negative impacts of RAS on targets from SDG1-2. The distribution of participant responses to whether RAS would have an impact on each target. Percentage values indicate the proportion of negative, neutral and positive scores. “Do not know” values were excluded. N(+) and n(-) indicate the total number of participants’ answers on the positive (+) and negative (-) impact of RAS on each target. The content of this publication has not been approved by the United Nations and does not reflect the views of the United Nations or its officials or Member States (https://www.un.org/sustainabledevelopment/).

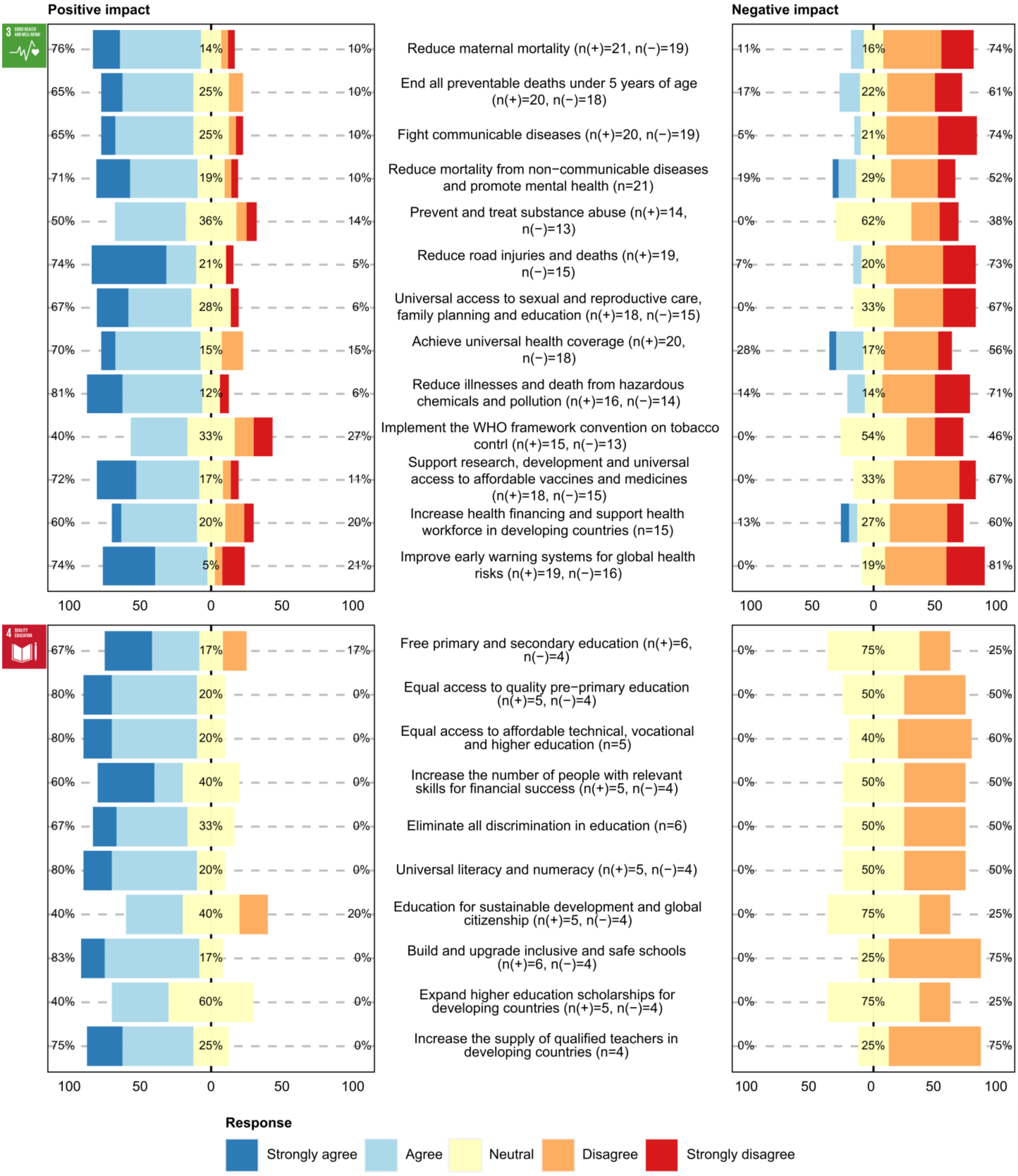

Supplementary Figure 4 Positive and negative impacts of RAS on targets from SDG3-4. The distribution of participant responses to whether RAS would have an impact on each target. Percentage values indicate the proportion of negative, neutral and positive scores. "Do not know" values were excluded. N(+) and n(-) indicate the total number of participants' answers on the positive (+) and negative (-) impact of RAS on each target. The content of this publication has not been approved by the United Nations and does not reflect the views of the United Nations or its officials or Member States (https://www.un.org/sustainabledevelopment/).

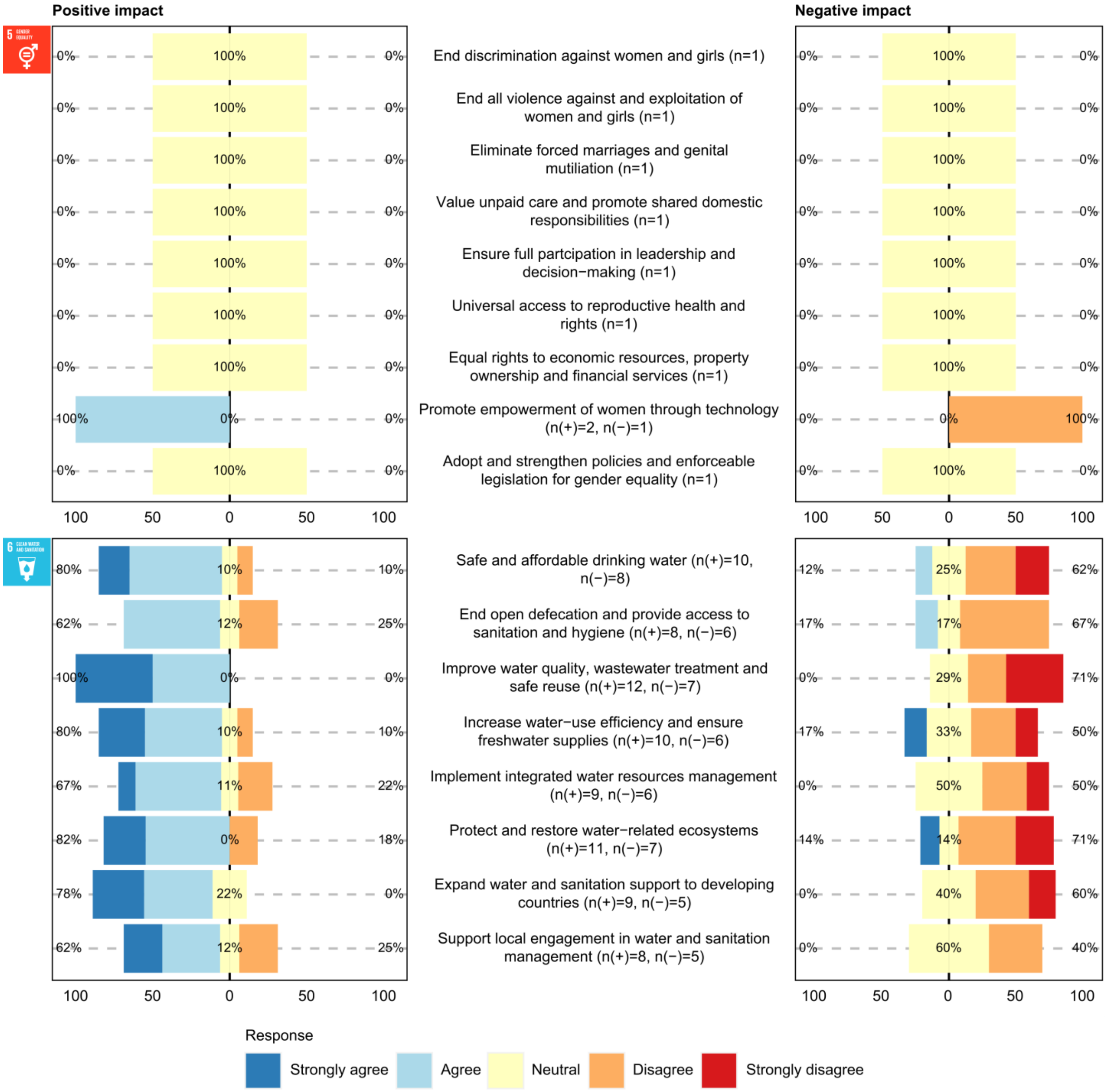

Supplementary Figure 5 Positive and negative impacts of RAS on targets from SDG5-6. The distribution of participant responses to whether RAS would have an impact on each target. Percentage values indicate the proportion of negative, neutral and positive scores. "Do not know" values were excluded. N(+) and n(-) indicate the total number of participants' answers on the positive (+) and negative (-) impact of RAS on each target. The content of this publication has not been approved by the United Nations and does not reflect the views of the United Nations or its officials or Member States (https://www.un.org/sustainabledevelopment/).

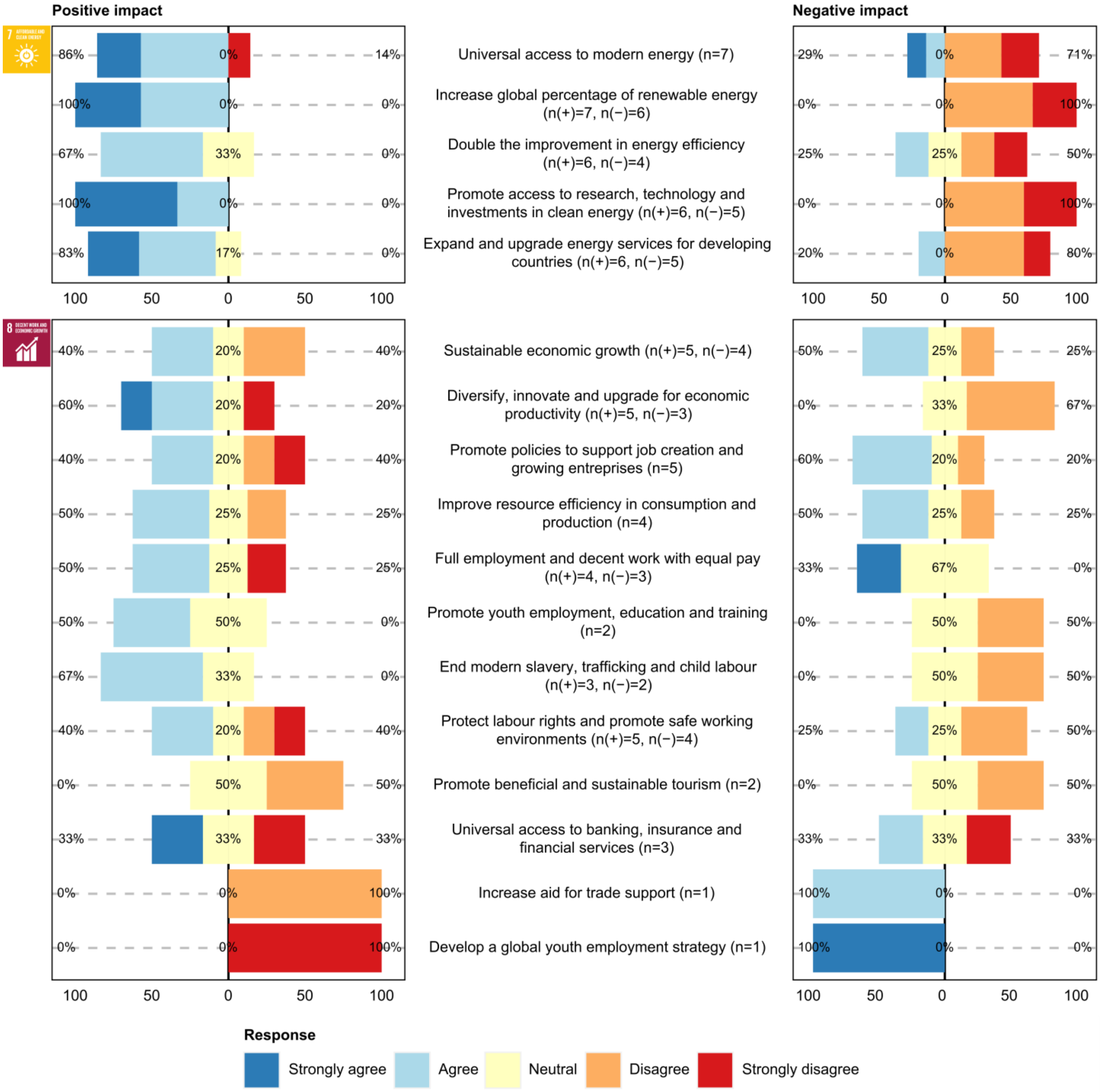

Supplementary Figure 6 Positive and negative impacts of RAS on targets from SDG7-8. The distribution of participant responses to whether RAS would have an impact on each target. Percentage values indicate the proportion of negative, neutral and positive scores. “Do not know” values were excluded. N(+) and n(-) indicate the total number of participants’ answers on the positive (+) and negative (-) impact of RAS on each target. The content of this publication has not been approved by the United Nations and does not reflect the views of the United Nations or its officials or Member States (https://www.un.org/sustainabledevelopment/).

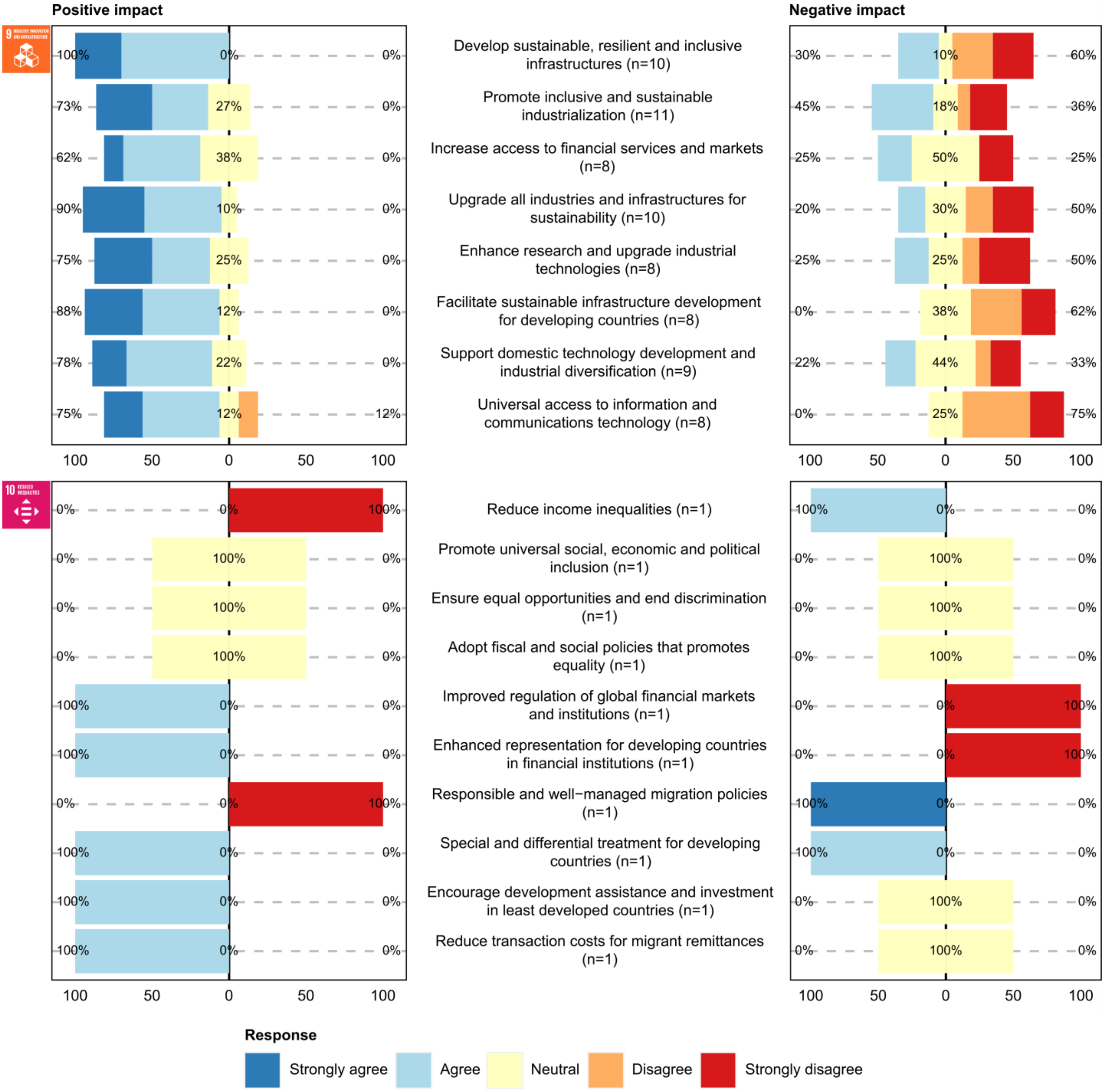

Supplementary Figure 7 Positive and negative impacts of RAS on targets from SDG9-10. The distribution of participant responses to whether RAS would have an impact on each target. Percentage values indicate the proportion of negative, neutral and positive scores. “Do not know” values were excluded. N(+) and n(-) indicate the total number of participants’ answers on the positive (+) and negative (-) impact of RAS on each target. The content of this publication has not been approved by the United Nations and does not reflect the views of the United Nations or its officials or Member States (https://www.un.org/sustainabledevelopment/).

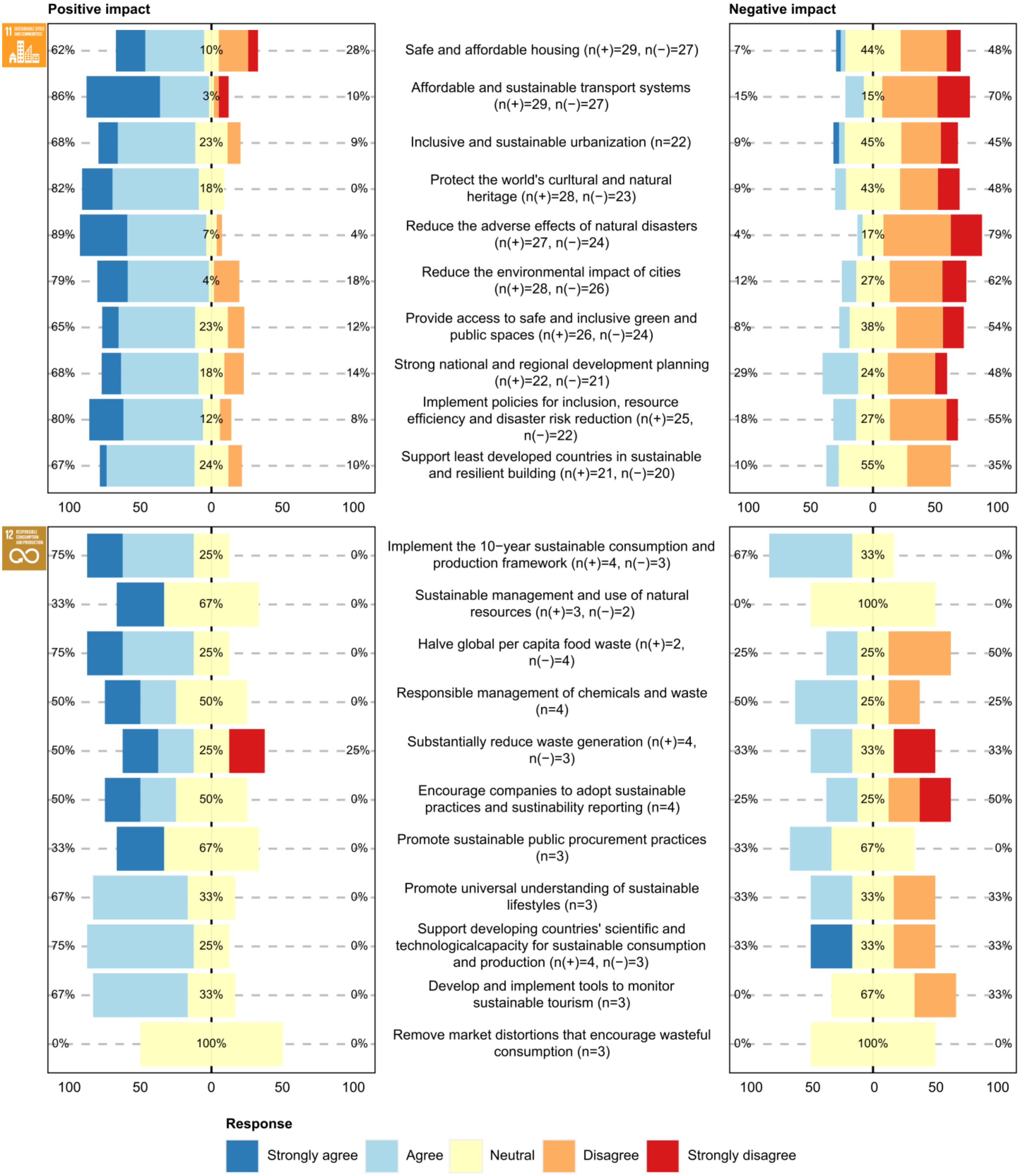

Supplementary Figure 8 Positive and negative impacts of RAS on targets from SDG11-12. The distribution of participant responses to whether RAS would have an impact on each target. Percentage values indicate the proportion of negative, neutral and positive scores. “Do not know” values were excluded. N(+) and n(-) indicate the total number of participants’ answers on the positive (+) and negative (-) impact of RAS on each target. The content of this publication has not been approved by the United Nations and does not reflect the views of the United Nations or its officials or Member States (https://www.un.org/sustainabledevelopment/).

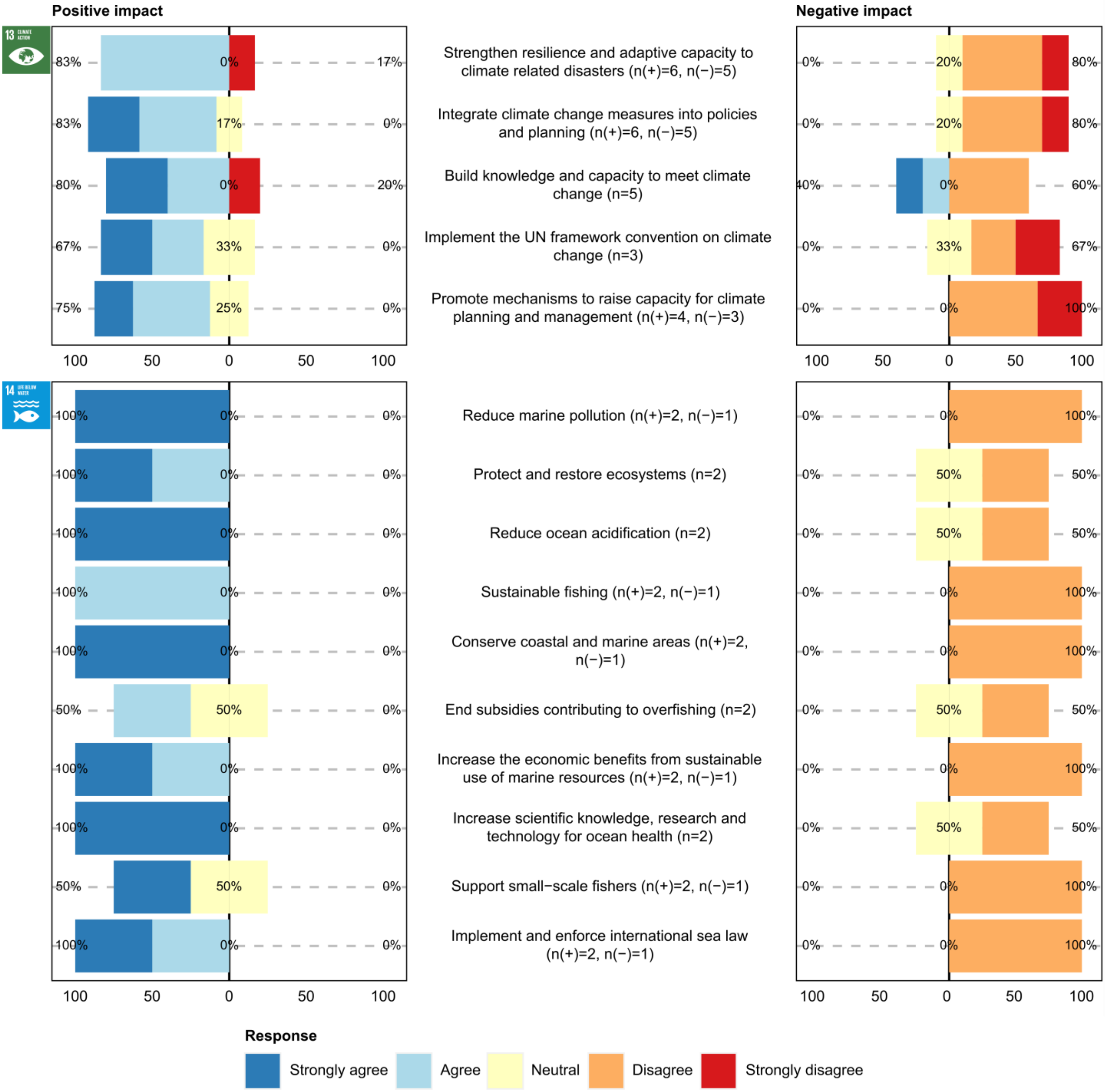

Supplementary Figure 9 Positive and negative impacts of RAS on targets from SDG13-14. The distribution of participant responses to whether RAS would have an impact on each target. Percentage values indicate the proportion of negative, neutral and positive scores. “Do not know” values were excluded. N(+) and n(-) indicate the total number of participants’ answers on the positive (+) and negative (-) impact of RAS on each target. The content of this publication has not been approved by the United Nations and does not reflect the views of the United Nations or its officials or Member States (https://www.un.org/sustainabledevelopment/).

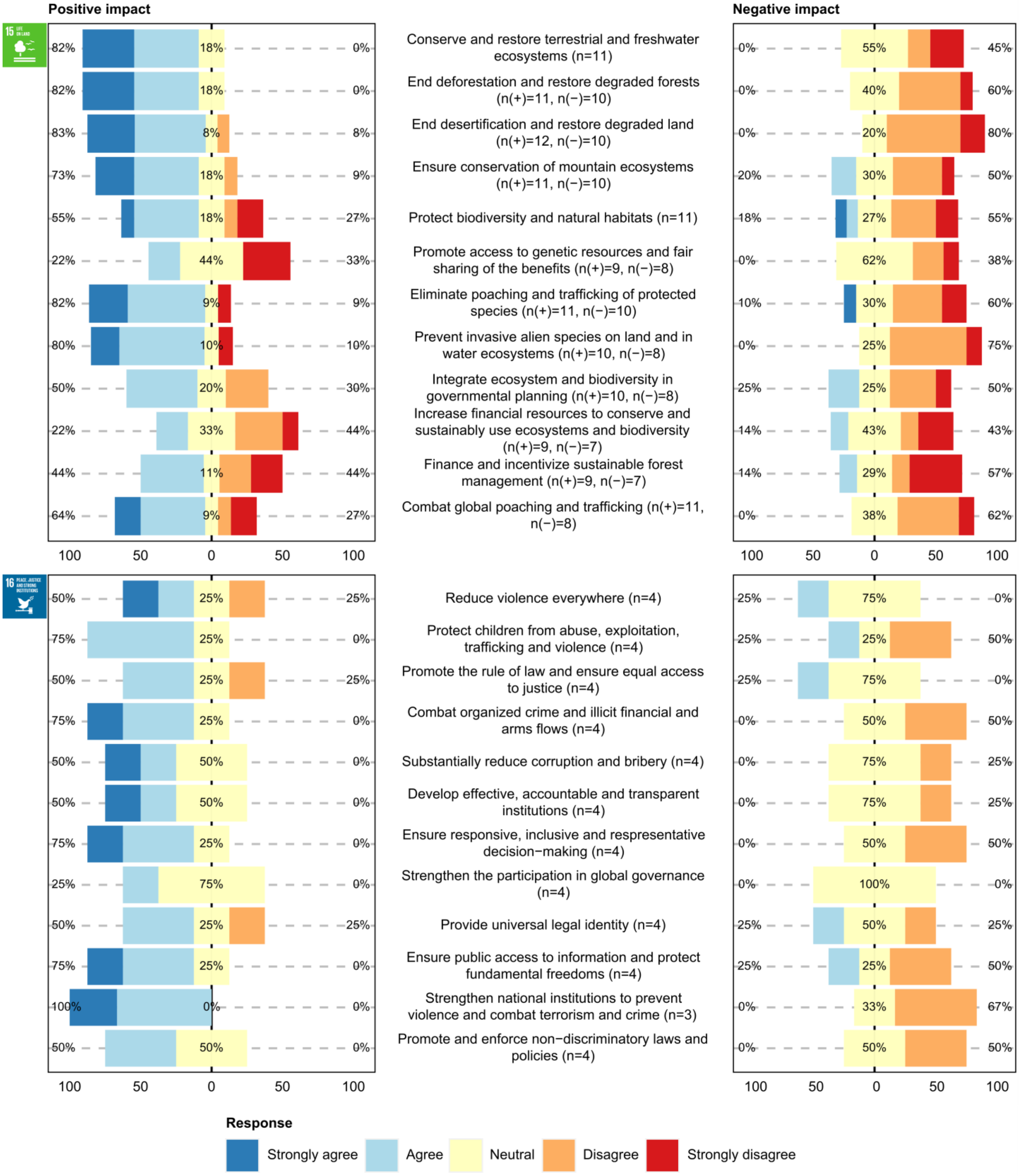

Supplementary Figure 10 Positive and negative impacts of RAS on targets from SDG15-16. The distribution of participant responses to whether RAS would have an impact on each target. Percentage values indicate the proportion of negative, neutral and positive scores. “Do not know” values were excluded. N(+) and n(-) indicate the total number of participants’ answers on the positive (+) and negative (-) impact of RAS on each target. The content of this publication has not been approved by the United Nations and does not reflect the views of the United Nations or its officials or Member States (https://www.un.org/sustainabledevelopment/).

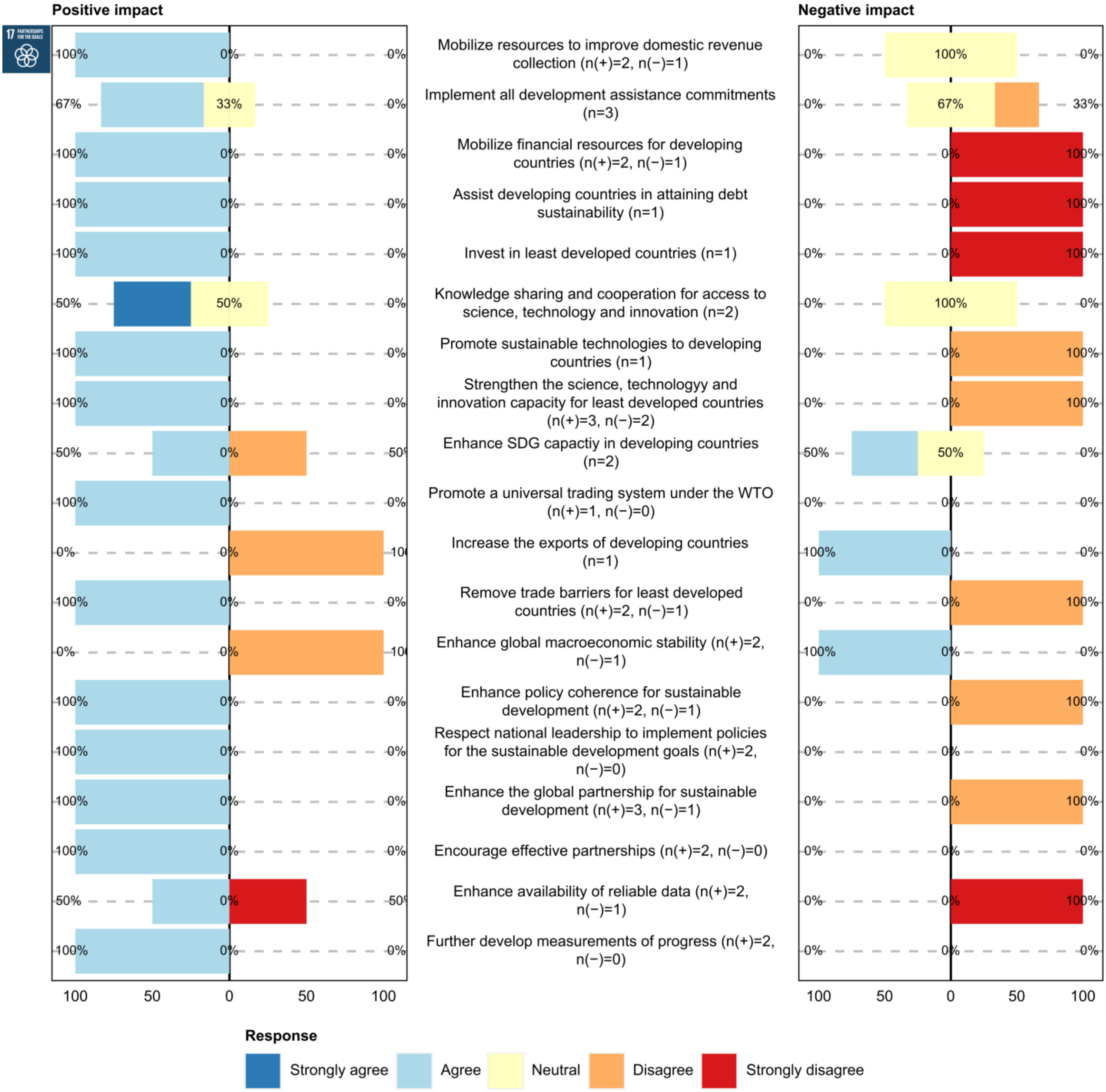

Supplementary Figure 11 Positive and negative impacts of RAS on targets from SDG17. The distribution of participant responses to whether RAS would have an impact on each target. Percentage values indicate the proportion of negative, neutral and positive scores. “Do not know” values were excluded. N(+) and n(-) indicate the total number of participants’ answers on the positive (+) and negative (-) impact of RAS on each target. The content of this publication has not been approved by the United Nations and does not reflect the views of the United Nations or its officials or Member States (https://www.un.org/sustainabledevelopment/).
